# Supplementary material for: “That You Just Know You’re Not Alone and Other People Have Gone through It Too.” Eating Disorder Recovery Accounts on Instagram as a Chance for Self-Help? A Qualitative Interview Study among People Affected and Self-Help Experts
Source: Int J Environ Res Public Health. 2022 Sep 9;19(18):11334. doi: 10.3390/ijerph191811334 (PMC9517556; doi:10.3390/ijerph191811334)
Supplement: Supplementary file 1 [file ijerph-19-11334-s001.zip › ijerph-1902719-supplementary.pdf]

Table S1. Interview guide.

Recovery account people		
**Topic**	**Main Questions**	**Follow up Questions**
**Introduction**	- Please introduce yourself and describe your relation to eating disorders.	- How did you come to create the Instagram account?
**Definitions**	- Please describe what you understand by *recovery* in relation to eating disorders?	
**Information**	- Please describe what you post?<br>- How do you choose what to post?<br>- Are there things you don't post? Which posts do you not share / publish?	- What do you pay attention to?<br>- What are taboo topics for you on Instagram?<br>- What would you never post?<br>- What are your experiences with emotions on Instagram?
**Usage**	- Please tell me how you use Instagram?	- Please describe how often you post news on your account and how often you are on other topic-related sites?<br>- How much time do you spend each day using Instagram (related to the topic of eating disorders)?<br>- Are there periods when you use Instagram more/less frequently?
**Patient exchange**	- Please describe the exchange with your followers?<br>- What do you appreciate about it? What is rather negative or problematic?	- What do you appreciate most?<br>- What are good experiences?<br>- Please describe your followers.<br>- What topics do you exchange about?<br>- Where do you see the limitations of Instagram?<br>- Is there any exchange beyond that?
**Effects**	- What do you notice about yourself when you use Instagram?	- What positive things do you notice?<br>- What, if anything, is also negative?<br>- What do you notice about yourself when you use it more/less often?
**Conclusions**	- What advantages do you see on Instagram in relation to your eating disorder?<br>- What disadvantages do you see?<br>- Please describe whether virtual exchange can replace real exchange for you? And, if yes/no, why?<br>- Please summarize where and how you think social media can help in the treatment of eating disorders?	

Self-help experts		
**Topic**	**Main Questions**	**Follow up Questions**
**Introduction**	- Please introduce yourself briefly and describe your relation to self-help.	
**Information**	- Please describe the role of knowledge transfer and information sharing in self-help?	- What standards are there in terms of the quality of information?<br>- What does the issue of "false information" look like in self-help?
**Patient exchange**	- Please tell me about the role of the exchange between the people affected.	- What makes the exchange so important for self-help?
**Self-help and social media**	- The topic of self-help on the internet: What experience do the organizations have in this area (NAKOS)?<br><br>The interview then focused on recovery accounts on Instagram:<br><br>- Please try to describe where you see similarities and differences to community self-help?<br>- Please describe which structures would have to be established in self-help so that social media would be a support / a gain?	- Where do you see chances for self-help?<br>- Where do you see risks for self-help?<br>- What can the use of internet/social media contribute / add? What is not possible?<br>- What advantages and disadvantages do you see with regard to the exchange of information on social media?<br>- What could Instagram in particular contribute to self-help? What is not possible?<br>- Why is social media not yet used in the context of self-help?
